# Frameless stereotactic brain biopsy and external ventricular drainage placement using the RONNA G4 system

**DOI:** 10.1093/jscr/rjac151

**Published:** 2022-05-31

**Authors:** Marina Raguž, Domagoj Dlaka, Darko Orešković, Anđelo Kaštelančić, Darko Chudy, Bojan Jerbić, Bojan Šekoranja, Filip Šuligoj, Marko Švaco

**Affiliations:** 1 Department of Neurosurgery, University Hospital Dubrava, Zagreb, Croatia; 2 Catholic University of Croatia, School of Medicine, Zagreb, Croatia; 3 Department of Surgery, School of Medicine, University of Zagreb, Zagreb, Croatia; 4 Faculty of Mechanical Engineering and Naval Architecture, University of Zagreb, Zagreb, Croatia

## Abstract

Robot-assisted stereotactic procedures are among the latest technological improvements in neurosurgery. Herein, to the best of our knowledge, we report a first external ventricular drainage (EVD) placement using the RONNA G4 robotic system preformed together with brain biopsy, all in one procedure. A patient was presented with progressive drowsiness, cognitive slowing, poor mobility and incontinent. Magnetic resonance imaging brain scans revealed multicentric process located in the basal ganglia right with extensive vasogenic edema and dilatated ventricular system. Using the RONNAplan software two trajectories were planned: one for brain biopsy on the left side and one for EVD implantation on the right side; the procedures went without complications. The RONNA G4 robotic system is an accurate neurosurgical tool for performing frameless brain biopsies and EVD placement. Further studies are needed in order to enroll a larger patient sample and to calculate the possible placement deviation, and to perform the comparison with other robotic systems.

## INTRODUCTION

Robot-assisted stereotactic procedures are among the latest technological improvements in neurosurgery. Lately, robotics has become relevant with increased use in several fields due to precision, reliability and spatial accuracy and dexterity, with improvements in the safety and efficacy of neurosurgical procedures [1, 2]. Robotic-assisted neurosurgical stereotactic procedures used widely are tumor biopsies, deep brain stimulation electrodes placement, stereoelectroencephalographic electrodes placement, external ventricular drainage (EVD) placement, endoscopy, etc. [1–9].

The RONNA G4, the fourth generation of the robotic neuronavigation system RONNA is regularly used as a standard neurosurgical tool for precise preoperative planning and frameless neuronavigation [3, 7, 8, 10].

Herein, to the best of our knowledge, we report the first EVD placement using the RONNA robotic system preformed together with brain biopsy, all in one procedure.

## CASE REPORT

**Figure 1:**
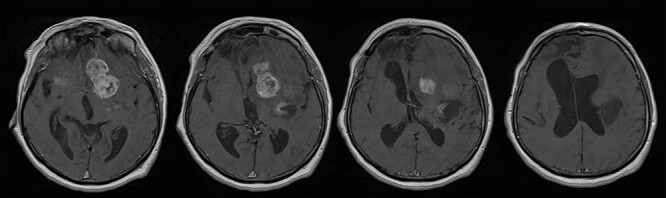
Brain MRI scans revealed an irregular expansive multicenter process located in the basal ganglia, frontobasal and in the area of the frontal operculum left and frontotemporal right with extensive vasogenic edema of the left frontal and parietal lobe and a compressive effect on the left lateral and III ventricle; after the administration of intravenous contrast, the process showed heterogeneous enhancement. Ventricular system was dilated with sign of transependymal edema.

A 65-year-old female patient was presented with progressive drowsiness, cognitive slowing, poor mobility and incontinent. In addition, the patient had sensorimotor dysphagia and right-sided hemiparesis. Initial computerized tomography (CT) and magnetic resonance imaging (MRI) brain scans revealed an irregular expansive multicenter process located in the basal ganglia, frontobasal and in the area of the frontal operculum left and frontotemporal right with extensive vasogenic edema of the left frontal and parietal lobe and a compressive effect on the left lateral and III ventricle. After the administration of intravenous contrast, the process showed heterogeneous enhancement (Fig. 1). Ventricular system was dilated with a sign of transependymal edema.

**Figure 2:**
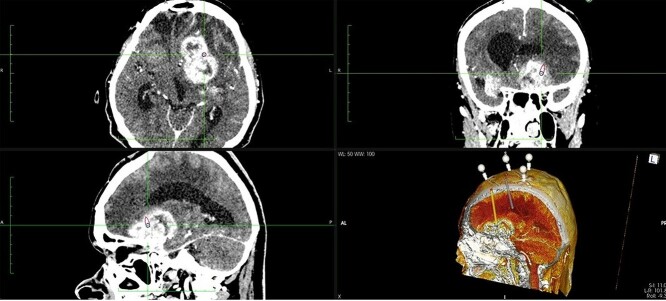
Two separate trajectories were planned using RONNAplan software, for brain biopsy and for EVD implantation. The plan is presented in three planes with 3d reconstruction model in right lower quadrant. Orange trajectory is for brain biopsy, whereas grey one is for EVD implantation.

**Figure 3:**
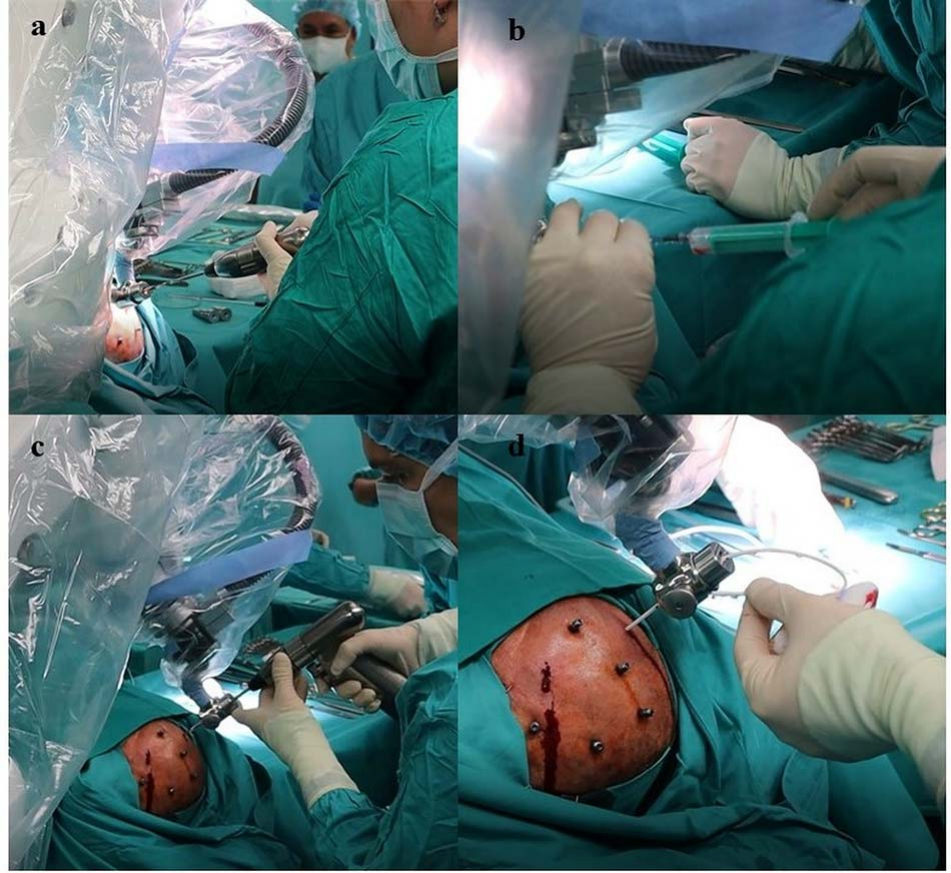
Frameless stereotactic brain biopsy and external ventricular drainage placement using the RONNA G4 system. (**A**) Using RONNA, skin perforation and twist drilling with a 4.5-mm drill and electrocoagulation of dura is done, as described in Dlaka et al. 2021. for frameless stereotactic brain biopsy, (**B**) obtaining tumor tissue for pathohistological analysis [7, 8]. Afterwards, the robot changed position to obtain proper position for EVD placement. (**C**) After skin perforation, twist drilling, and electrocoagulation, (**D**) an EVD catheter was placed in right lateral ventricle and clear cerebrospinal fluid was obtained under elevated pressure.

The patient underwent frameless brain biopsy using the RONNA robotic system as described previously obtaining tumor tissue for pathohistological analysis [3, 7, 8]. Using RONNAplan software two trajectories were planned, one for brain biopsy on the left side and one for EVD implantation in the right side (Fig. 2). After skin perforation, twist drilling with a 4.5-mm drill, and electrocoagulation, an EVD catheter was placed in the right lateral ventricle and clear cerebrospinal fluid was obtained under elevated pressure (Fig. 3). The total operative time, including trajectory planning and surgical procedure, was 65 min.

The immediate postoperative course was uneventful. The brain CT performed on the first postoperative day confirmed satisfactory finding with EVD catheter in proper position. Due to clinical improvement, the ventriculoperitoneal (VP) drainage system was implanted on the ninth postoperative day. Tumor tissue samples acquired during the surgery corresponded to glioblastoma grade IV. Hence, the patient was appointed towards further oncologic treatment.

## DISCUSSION

Modern robotic systems are able to perform safe, highly accurate, highly reproducible and efficient intracranial targeting. Our current system RONNA G4 ensures highly accurate frameless stereotactic procedures [7, 8, 10]. In comparison with commercially available robotic systems such as ROSA, Neuromate, SurgiScope, Renaissance, etc. the RONNA system is still an experimental system. We use RONNA routinely for frameless stereotactic biopsies with high precision. Due to the mentioned accuracy we decided to go a step further and used RONNA for EVD placement. EVDs provide lifesaving treatments in patients with traumatic brain injury, hemorrhage, etc. Traditional free-hand EVD placement has been associated with a relatively frequent occurrence of complications, such as risks of intracerebral hematoma, and improper placement.

Robot-assisted ventricular catheter placement either as a part of VP shunting or as a EVD placement is a promising approach. A trajectory could be calculated by taking into account ventricular size, shape and other characteristics as well as entry point, avoiding blood vessels, similar like in biopsy trajectory planning. Since Lollis and Roberts study in 2008, where 16 patients requiring EVD received successful ventricular cannulations on first pass, a catheter placement using number of robot-assisted systems was done [1, 2, 4–6, 11]. Moreover, some authors design a prototype of the robotic navigation system to allow surgeons who do not have explicit neurosurgical training to place EVDs [9].

The clinical peculiarities should be emphasized. There was an absolute indication for a biopsy and EVD placement, and both parts were done in one procedure. No technical problem was observed regarding the complexity of planning two trajectories. The surgical technique was modified for EVD placement procedure. When more trajectories are needed, a minimum increase in time is needed for extra trajectory compared to the classical approach. Although EVD placement alone is frequently a routine procedure and does not need use of robotic technology, when performed in combination with other procedure such as brain biopsy, it offers significant improvement in accuracy.

Given the literature review, there are several advantages in using RONNA for EVD placement. For traditional EVD placement, skin incision followed by 11-mm diameter burr hole, and dura incision is done prior to placement. Using RONNA, skin perforation and twist drilling with a 4.5-mm drill and electrocoagulation of dura is done, as described previously [7, 8]. The small diameter reduces the brain exposure and decrease intraoperative bleeding; cerebrospinal fluid leaks are avoided and therefore brain shift, and possibility for infection was minimized. Furthermore, following the trajectory plan the EVD is placed accurately, in one attempt. Robot-assisted EVD placement enables careful preoperative study and trajectory optimization, minimizing the risks to bridging veins and sulcal vessels. In addition, the risk of complications from passing through the brain tissue damaging the basal ganglia, corticospinal tract, etc. and causing dysfunction is minimized [4–6, 9].

An increase in neurosurgeon’s experience over time is associated with shorter procedure duration. In previous study, the duration of frameless brain biopsy using RONNA system was ~70 min [8]. In this case, we performed brain biopsy and EVD placement in 65 min. Indeed, we are reducing the duration time of the procedure continuously, as one of the important factors is neurosurgeon’s previous experience in robotic procedures [7, 8].

RONNA robotic system was shown as an accurate neurosurgical tool for performing frameless brain biopsies and EVD placement, even in a single procedure, with shorter operation time resulting in avoiding operative risk factors, EVD implantation using twist drill, accurate two and more trajectory planning strategy from technical and clinical point of view, and minimally invasive approach resulting in decreased possibility for postoperative complications. Further studies are needed in order to enroll a larger patient sample and to calculate the possible placement deviation, and to perform the comparison with other robotic systems.

## COMPLIANCE WITH ETHICAL STANDARDS

The patient has given an informed consent for participation in this paper.

## CONFLICT OF INTEREST STATEMENT

On behalf of all authors, the corresponding author states that there is no conflict of interest.
